# Death anxiety scale (DAS): internal structure and factorial invariance in Peruvian adults

**DOI:** 10.12688/f1000research.143167.2

**Published:** 2024-11-25

**Authors:** Carlos De La Cruz-Valdiviano, Aldo Bazán-Ramirez, Lincol Olivas-Ugarte, Juan Quijano-Pacheco

**Affiliations:** 1 Piura, Cesar Vallejo University, Piura, Piura 20001, Peru; 2 Apurímac, Jose María Arguedas National University, Andahuaylas, Andahuaylas CP 03701, Peru; 3 Lima, Cesar Vallejo University, Lima Norte, Los Olivos, Lima CP 15314, Peru; 4 La Libertad, Cesar Vallejo University, Trujillo, Trujillo 13001, Peru

**Keywords:** death anxiety, validity, reliability, fairness/equity, adults.

## Abstract

**Background:**

Studies have this reported the presence of death anxiety, ranging from near-death experiences to physically ill people, to a simple thought about death, as well as when it is associated with states of loneliness. The Templer’s Death Anxiety Scale (DAS), based on Eysenk’s incubation theory, has been adapted and validated in different contexts and is still insufficient in countries such as Peru. This study aimed to determine the psychometric properties of the DAS, its internal structure, and factorial invariance.

**Methods:**

An online scale was used in 1248 Peruvians aged between 18 and 70 years (M= 27.37, SD= 11.29) from all regions. DAS was validated using exploratory factor analysis (EFA), confirmatory factor analysis (CFA), convergent validity, measurement invariance, and internal consistency (reliability).

**Results:**

Psychometric properties were found with adequate values in its structure and validity based on the internal structure through exploratory factor analysis, where it was found that the components of the scale are interrelated and the data matrix is factorizable. model of three specific factors and a general factor is presented, which is consistent with the theory and has practical utility, revealing adequate statistical values that reflect acceptable levels of reliability. Finally, there was fairness according to sex and age group of the scale by means of factorial invariance analysis.

**Conclusions:**

Our results indicate adequate psychometric properties and facilitate a better data collection process for future research.

## 1. Introduction

Death is a universal human fact, ^1^ and people experience death anxiety in different ways, and the way they express it is usually particular according to sociodemographic variables such as age, sex, religion, among others, or psychological variables, such as self-esteem, personality, mood, or coping styles ^1^^,^^2^ In this regard, other authors ^3^ pointed out that there are two main variables: mental health and near-death experiences (accidents or terminal illness) that influence the degree of death anxiety. However, Tomás-Sábado and Gómez-Benito ^2^ argued that death anxiety occurs both in people who are physically ill and in those who think about death.

In this context, in 2020, according to the Pan American Health Organization/World Health Organization, ^4^ the spread of SARS-Cov-2 was declared a public health emergency, known worldwide as COVID-19. Orús ^5^ mentioned that about 6.3 million people died due to this virus as of June 12, 2022. Due to its appearance, several measures have been taken in this regard, and people’s daily lives have been altered in different dimensions, such as work performance, family and social ties, educational activities, economic instability, and health conditions, which are factors that become indicators of anxiety among other psychological repercussions. According to the PAHO/WHO ^6^ during the first year of the pandemic, the prevalence of anxiety or fear of death has increased by 25% worldwide, with young people being part of the most affected population. Thus, nowadays, all people live in a very different context to the one they used to know and thus find different reactions to it, as life conditions are altered, which is why many people have suffered from this silent disorder that is anxiety, which is a recurrent topic in research since its features are visible in society. ^7^

A study conducted by the University of Bristol found that the number of young people with anxiety increased from 13% to 24%. ^8^ Thus, death anxiety is a reaction generated by the sensation of risk, whether real or imaginary, that originates from environmental stimuli. ^9^ Taking as a reference what has already been mentioned, it is understandable that when the routine cycle of people’s lives is altered, these have an impact on more internal levels such as ways of thinking, habits that have changed, and the intensity of emotions, which is directly reflected in the behavior that in the end is the manifestation of how each person has internalized this situation. In Metropolitan Lima, where the main districts affected by the pandemic are located because of the high level of contagion and the numerous deaths that have occurred, the general population is taken as a reference for the pandemic reality in order to determine anxiety as a result of this context. ^10^ It is, therefore, necessary for specialists to have a reliable instrument with which to obtain real results and thus be able to perform an optimal screening in order to analyze the levels of anxiety in the Peruvian population.

The Death Anxiety Scale (DAS) created by Templer ^11^ and authorized by its authors in the Mexican version ^12^ was used to measure these variables. Therefore, given that an instrument would be very helpful in exploring what has already been explained, the following question was formulated: What is the evidence of validity, reliability, and fairness of the death anxiety scale (DAS-1) in Peruvian adults, 2022? At the methodological level, the study provides rigor in terms of validity, reliability, equity, and psychometric characteristics of an instrument for Peruvian adults. Practically, it encourages professionals who deal with mental health to develop innovative research where standardized, valid, and reliable instruments need to be applied as a screening prior to specialized care. This results in death anxiety assessments relevant to the cultural context in which they are applied. Although Templer’s DAS has resisted the test of time as a commonly used index to capture the conscious experience of death anxiety, a continued evaluation of how the DAS translates in specific countries is required with an assessment in relation to other death construct tools, ^13^ such as the population of adults aged 18 to 70 years, which in Peru make up the majority of the population ^14^ that will lead the way to the country’s development. At the same time, it is relevant because it focuses on one of the most important topics, such as anxiety that the Peruvian adult population may be related to the post-pandemic context. Likewise, it will serve as a background for prospective studies whose objective is to study the same variable, and finally, its purpose is to support early intervention for events that could have serious consequences for this population.

Psychometric tests provide measurable and objective data that provides an overview of the performance of an individual or population. This scale provides a degree of scientific reliability and objective recruitment process. In addition, because it is a standardized test, it reflects accurate information. Therefore, the general objective was to analyze the psychometric properties of the Death Anxiety Scale (DAS) in the Peruvian adult population. Accordingly, following a sequential order, the specific objectives were formulated as follows: 1) to verify the metric quality of the items, and 2) to examine the evidence of validity based on of the internal structure, 3) to examine the evidence of reliability, and finally 4) to examine the evidence of fairness according to sex and age group of the DAS Scale in Peruvian adults.

### 1.1 Background and Eysenck’s theory of incubation

Rivera and Montero ^12^ carried out an adaptation in older adults and university students from Mexico; three dimensions were obtained in each group, and internal consistency was.86 and.83, respectively. Lopez-Castedo et al. ^15^ analyzed patients with ischemic heart disease in Spain, finding positive correlation corrected item-total correlation in all items (0.32 and 0.54). Four factors were identified that together explained 51.85% of the variance in the data, while the reliability values were high (0.77). Resett et al ^16^ examined 859 adults in Argentina. Factor analyses showed a bifactor structure with higher adequacy than a unifactor structure. These findings allow us to argue that the scale showed evidence of validity and reliability (.80) for that country.

In Peru, few studies have been conducted on death anxiety; in Trujillo, 50 hospitalized patients found that it is negatively related to mental adjustment to cancer; that is, the greater the mental adjustment to cancer, the lower the anxiety about death. ^17^ Peñaloza ^18^ analyzed the content validity of health personnel in Lima. AFC explains the three-factor model. The correlation with PIL-TEST was strongly negative (rho = − .630). Reliability by internal consistency through α and **ω** = .95. Analysis of invariance by sex indicated that the scores differed between men and women. In addition, Graus ^19^ in older adults in Trujillo by confirmatory factor analysis, and reliability by internal consistency (.93) was very good. Rodriguez, ^20^ in older adults in Chimbote, obtained reliability through **ω** and gave.81 for fear of agony or illness, .79 for fear of life coming to an end and.90 for fear of death. The results indicated that the scores ranged from acceptable to very good.

Although the first studies covered clinical populations, there are also studies in the general population when considering the influence of sociocultural factors, ^21^ where anxiety about death can vary significantly between countries and cultures, depending on elements such as religious beliefs, social norms and exposure to conflict contexts. This aspect is particularly relevant for the Peruvian population, given the cultural diversity and sociocultural values. Likewise, anxiety about death has been investigated in young and middle-aged adults, at the same time, studies such as those by Abdel-Khalek and Neimeyer ^22^ have shown that anxiety about death is not exclusive to clinical populations or older adults. Also, gender differences have been analyzed in the manifestation of anxiety about death in the general population, ^22^ drawing attention to the fact that women tend to report higher levels of anxiety about death than men. Finally, recent studies have explored the relationship between anxiety about death and psychological well-being in the general population. According to Abdel-Khalek and Neimeyer, ^22^ death anxiety is inversely related to subjective well-being, this finding is particularly relevant to emphasize the importance of adequately measuring death anxiety in the general population.

### 1.2 Anxiety

For RAE, ^23^ anxiety is a situation of mood agitation, usually a state of fear associated with various diseases. According to Limonero ^24^ fear indicates an expected reaction to the threat posed by anxiety-provoking events. It refers to a target that causes a bisector of discomfort, such as instability, restlessness, and fussiness, in the face of a surprise. Anxiety is a state of unconscious emotion, such as undefined despair.

Hamilton ^25^ notes that fear can be present within normality or illness. Similarly, anxiety presents with a variety of symptoms, including cardiovascular pain, abdominal pain, dyspnea, and mental, cognitive, or subjective symptoms. The present work is based on Eysenck’s incubation theory. ^26^ This, in turn, is based on classical test theory. ^27^

## 2. Methods

### 2.1 Participants

The sample consisted of 1248 Peruvian adults between 18 and 70 years of age (Arithmetic Mean [M] = 27.37, Standard Deviation [SD] = 11.29) from all regions of Peru. To select this sample, non-probabilistic convenience sampling was used, taking into account the corresponding inclusion and exclusion criteria. ^28^^–^^30^ According to Comrey and Lee, ^31^ a sample size of at least 100 is considered poor, and a sample size greater than or equal to 1000 is considered excellent.

The following inclusion criteria were established for the selection of participants: adults between the aforementioned ages residing in the Peruvian territory; those who would respond favorably to informed consent; those who had completed the responses to the instruments in Google Forms through the online link that was sent to them. Once the responses to the Google Form were obtained, the number of subjects was 1288. However, after careful review, 40 individuals were eliminated (19 said they were retired, widowed, or divorced, all under 20 years of age; 10 were born or lived in another country; 11 did not complete their answers) to avoid possible bias in the information. Those between 18 and 30 years of age stand out (39.9% men and 30% women), and in terms of place of residence, 40.1% of men and 36.5% of women live in the Department of Lima.

### 2.2 Measurement

Self-administered online surveys were conducted using a written questionnaire and distributed by e-mail. ^32^ The Death Anxiety Scale (DAS -1) was created by Donald I. Templer in the United States in 1970, ^11^ where its application was individual and collective. Later, in 2002, it was adapted and translated into Spanish by Tómas Sábado and Gómez-Benito; in 2010, it was adapted and translated by Rivera y Montero into Mexican Spanish. In Peru, adaptation was given in 2016 in Trujillo by Graus. ^19^ The time to consider is 10 to 15 min, while its administration is from 18 to 85 years of age. It is composed of 15 items with four Likert-type options. ^73^ The items of this scale consider statements related to concepts related to death and their emotional repercussions on people, considering their impact on them. Initially, there were true and false response options; however, the dichotomous alternatives were modified by alternatives on a Likert scale with the objective of allowing a better variability in the responses that reflects what people feel or think in relation to death, thus improving the internal consistency of the scale itself. Scores were obtained using sums or averages. The participants provided four types of answers on a Likert scale: never or almost never (1), sometimes (2), most of the time (3), and all the time (4), with a minimum score of 15 (low anxiety) and 60 (high anxiety). In dimension I: Fear of agony or pathology, the minimum score was 5, and the highest was 20. In dimension II: Fear of life coming to an end, the minimum score was 7, and the highest was 28. Finally, dimension III: Fear of death, the minimum score is 3, and the highest is 12.

The instrument has psychometric characteristics verified in the United States and Spain, with Cronbach’s alpha fluctuating between .76 and .87 and a fairly good retest security, r = .71 and r = .84. ^12^ Its construct value has been extensively studied, and its partnership has been defined with many psychological variants, such as depression and anxiety (r = .38 and r = .48, respectively). ^12^^,^^33^ However, studies of its factorial composition are more contradictory in the analysis, sample, and cultural setting in which it is used.

The three-factor Death Anxiety Scale is based on a rigorous empirical analysis that reflects the underlying structure of the data and is in line with previous cross-cultural studies that have identified multidimensional structures. This conceptualization allows for a more comprehensive and nuanced assessment of death anxiety in the Peruvian population, capturing specific aspects that may not be evident in a unidimensional structure. The results of Sharif Nia et al. ^13^ and Zuccala et al. ^34^ support the need to adapt the DAS scale according to the cultural and sociodemographic particularities of each sample.

### 2.3 Procedure

To carry out the study, the necessary permission was requested from the author in order to be able to measure the variable under analysis. Then, the online survey form was disseminated through the Google Forms application in which informed consent, sociodemographic data, and the measurement instrument were included, and the link to the form was sent to individuals who met the requirements of the inclusion criteria for their participation. They were applied through social networks, such as Facebook and WhatsApp. They were also sent by electronic correspondence, prioritizing the authorization of the same for execution, emphasizing anonymity, privacy, and voluntary collaboration. Finally, the responses of individuals who did not meet the sample profile were not considered, and the exploration was carried out correctly using statistical programs.

### 2.4 Ethical aspects

In accordance with the World Medical Association (WMA) regarding the Declaration of Helsinki, ^35^ specifically related to medical research on human beings, care was taken to consider primarily the health of the subject, precisely this work is oriented to favor conditions that promote their welfare (avoiding damage to their health), while ensuring and promoting satisfaction with the life and rights of those, with the purpose of contributing to screenings that facilitate diagnoses, preventive interventions and quality of treatments. The protocol was submitted for consideration, comment, guidance and approval by the Research Ethics Committee of the Cesar Vallejo University (Dictamen 070-CEI-EPM-UCV-2022) ^73^^,^^74^ on September 28, 2022, after which the research could be carried out The participants were previously informed about the nature of the questionnaires, the importance of the study and the guarantee of their anonymity, then, regarding informed consent, the participants responded to their agreement via online. ^36^ Likewise, the study was carried out taking into account the authors cited, so that any type of plagiarism was avoided.

### 2.5 Statistical analysis

Once the survey application was finished, the participants’ responses were downloaded into a Microsoft Excel spreadsheet to prepare the database. This information was then exported for statistical processing using the free program, RStudio version 4.3. The data were analyzed as follows: First, preliminary statistical analysis of the items was performed using the psych package. ^37^ Statistics such as the mean (M), standard deviation (SD), coefficients of asymmetry (g1 = [-1.5; 1.5]), kurtosis (g2 = [-1.5; 1.5]), ^38^^,^^39^ corrected homogeneity index (CHI ≥ .30), ^40^ and communalities (h2 ≥ .40) ^41^ were examined. Additionally, the polychoric correlation matrix (|r|= [.30; .90]) was extracted. ^42^^,^^43^

Second, exploratory factor analysis (EFA) was performed using the psych and parameter package. ^44^ The polychoric correlation matrix was used as input, with the purpose of determining the underlying structure of the DAS Scale. Previously, the assumptions of the EFA were verified: matrix determinant (|A| ≈ 0), Kaiser-Meyer-Olkin sample adequacy test (KMO ≥ .80), and Bartlett’s test of sphericity (p< .001). ^45^ The following extraction methods were used: parallel analysis, optimal coordinates, and the Kaiser-Gutman rule. Likewise, the method of minimum residual estimation (Minres) was applied in combination with the oblimin rotation method. ^46^ Finally, factor loadings (λ ≥ .30) and interfactorial correlations (φ ≥ .50) were examined. ^43^^,^^47^^–^^49^

Third, confirmatory factor analysis (CFA) was performed using the Lavaan package. ^50^ The polychoric correlation matrix was used as the input because the data did not correspond to continuous variables with a normal distribution. ^51^ In addition, the Weighted Least Squares with mean and variance adjusted variance (WLSMV) estimator was applied due to the ordinal nature of the items. ^52^^,^^53^ Additionally, different factor structures were tested, as this is a good practice in psychometric studies. ^54^ Different fit indices were used for model evaluation, such as chi-square (χ ^2^, p < .05), Comparative Fit Index (CFI ≥ .94), Tucker-Lewis indices (TLI ≥ .94), standardized root mean square residual (SRMR ≤ .08), Root Mean Square Error of Approximation (RMSEA ≤ .07) ^55^ and RMSEA confidence intervals (Li ≤ .05 and Ls ≤ .10). In addition, because χ ^2^ is sensitive to sample size, the chi-square ratio over degrees of freedom (χ ^2^/gl ≤ 5) was evaluated. ^56^

In addition, specific indices for the bifactor models were examined. Likewise, the analysis of the indexes of the second-order model was considered, for which a Schmid-Leiman transformation ^57^ was performed, allowing the variance of each factor to be estimated, ^58^ both of which were processed using the EFA tool package. ^59^ The following were considered as indicators of: unidimensionality: The conjunction of the magnitudes of the hierarchical **ω** coefficient for the overall factor (ωH > .80), ^60^ of the hierarchical omega coefficient for the specific factors (ωhs ≥ .30) (Smits et al., 2014), ^61^ the construct replicability coefficient (H > .90), ^62^ common variance explained (ECV >.60), and percentage of uncontaminated correlations (PUC < .80). ^60^ Finally, the percentage variance of the factors (ωH2, ωhs2) was calculated as evidence of the instrument’s explanatory power. ^47^

Fourth, evidence of reliability was examined using the internal consistency method, quantifying its magnitude with the omega coefficient (ω) for multidimensional scales, ^63^ appropriate for congeneric measures, ^64^^,^^65^ and values ≥ .80 ^66^ were taken as the conventional cut-off point. Fifth, evidence of equity was evaluated by means of a factorial equivalence analysis according to sex and age group, ^67^ using the semTools package. ^68^ Four progressive levels of invariance were considered: 1) configural invariance (unrestricted), 2) metric invariance (factor loadings), 3) strong invariance (loadings and intercepts), and 4) strict invariance (loadings, intercepts, and residuals), taking the changes in CFI (ΔCFI ≤ .010), RMSEA (ΔRMSEA ≤ .015), and SRMR (ΔSRMR ≤ .030) ^69^^,^^70^ to determine the interpretability of the items by the examinees ^71^ and the equivalence of the scores obtained in the test, regardless of the participants’ membership. ^72^

## 3. Results

### 3.1 Statistical analysis of the items of the DAS Scale

As Table 1 shows, for the most part, the DAS Scale items meet the parameters previously established to claim that the data matrix can be reduced to a smaller set of latent factors: the magnitudes of the correlations|r|= [.30–.90], corrected homogeneity index (CHI ≥ .30), and communalities (h2 ≥ .40). Therefore, the metric quality of the DAS items was verified for inclusion in the AFE.

**Table 1.** Statistical analysis of the items of the DAS.

Items	M	DE	g ^1^	g ^2^	CHI	h ^2^	Matrix of polychoric correlations														
							A1	A2	A3	A4	A5	A6	A7	A8	A9	A10	A11	A12	A13	A14	A15
A1	1.62	0.66	0.97	1.27	.69	.53	1	−	−	−	−	−	−	−	−	−	−	−	−	−	−
A2	1.71	0.71	0.85	0.80	.54	.33	.50	1	−	−	−	−	−	−	−	−	−	−	−	−	−
A3	1.45	0.65	1.41	1.93	.67	.50	.58	.40	1	−	−	−	−	−	−	−	−	−	−	−	−
A4	1.68	0.72	0.95	0.80	.57	.35	.39	.19	.41	1	−	−	−	−	−	−	−	−	−	−	−
A5	1.68	0.70	0.95	1.08	.74	.61	.79	.48	.66	.47	1	−	−	−	−	−	−	−	−	−	−
A6	1.80	0.76	0.87	0.71	.66	.48	.45	.34	.43	.53	.52	1	−	−	−	−	−	−	−	−	−
A7	1.57	0.64	0.94	0.91	.77	.65	.65	.54	.69	.44	.68	.55	1	−	−	−	−	−	−	−	−
A8	2.17	0.86	0.46	-0.36	.56	.33	.34	.37	.26	.37	.33	.41	.38	1	−	−	−	−	−	−	−
A9	2.00	0.84	0.73	0.16	.72	.56	.54	.45	.47	.48	.59	.57	.54	.47	1	−	−	−	−	−	−
A10	1.57	0.74	1.30	1.45	.58	.37	.37	.38	.40	.34	.42	.35	.51	.42	.47	1	−	−	−	−	−
A11	1.56	0.69	1.25	1.72	.64	.45	.46	.31	.41	.46	.49	.59	.53	.37	.58	.43	1	−	−	−	−
A12	2.00	0.89	0.77	-0.03	.59	.36	.37	.38	.34	.32	.36	.43	.41	.63	.45	.43	.38	1	−	−	−
A13	1.54	0.68	1.23	1.61	.55	.32	.35	.24	.45	.33	.38	.46	.42	.32	.40	.34	.38	.37	1	−	−
A14	1.77	0.88	1.01	0.29	.51	.28	.39	.21	.46	.36	.38	.32	.43	.26	.39	.29	.35	.31	.40	1	−
A15	1.72	0.81	1.08	0.83	.60	.38	.36	.41	.41	.35	.42	.39	.46	.48	.40	.44	.36	.48	.41	.37	1

Note: M = mean; SD = standard deviation; g1= asymmetry coefficient; g2 = kurtosis coefficient; CHI = corrected homogeneity index; h2 = commonality.

### 3.2 Construct validity

As shown in Table 2, the AFE assumptions were previously verified: matrix determinant = .001, KMO test = .92, and Bartlett’s test of sphericity was statistically significant (p < .001), indicating that the variables are interrelated and that the data matrix is factorizable. Therefore, the procedure was repeated. The 15 items were then grouped into three factors with a total cumulative variance of 53.3%. Furthermore, the magnitudes of the interfactorial loadings (ϕ) [.40, .50] were consistent with an oblique three-factor correlated model. However, two items were discarded: item A9 (“I am afraid of dying a painful death”) for presenting very similar factor loadings in more than one factor (F1 = .32, F2 = .25, F3 = .33), and item A14 (“I am horrified to see a corpse”) for presenting low factor loadings (λ <.30). Consequently, we proceeded to run the CFA with only the other 13 remaining items.

**Table 2.** Exploratory factor analysis of the DAS.

Items	*λ*F1	*λ*F2	*λ*F3
A5	.864	-.074	.081
A1	.837	-.024	-.007
A7	.721	.091	.103
A3	.704	-.055	.143
A2	.590	.340	-.288
A8	-.095	.806	.080
A12	.010	.736	.051
A15	.200	.484	.072
A10	.260	.384	.085
A4	.101	.096	.561
A6	.144	.177	.561
A11	.187	.149	.500
A13	.164	.190	.347
A9	.320	.255	.330
A14	.266	.081	.286
Interfactorial loadings (ϕ)			
F1	1	−	−
F2	.543	1	−
F3	576	.457	1
Kaiser-Meyer-Olkin test	.92		
Bartlett's test of sphericity	χ ^2^(gl) = 456.31(14), *p* < .001		
Matrix determinant	.001		
% Explained variance	25.2	25.2	25.2
% Accumulated variance	25.2	25.2	25.2

Note: χ ^2^ = Chi-square ratio, gl = degrees of freedom.

**Figure 1.**
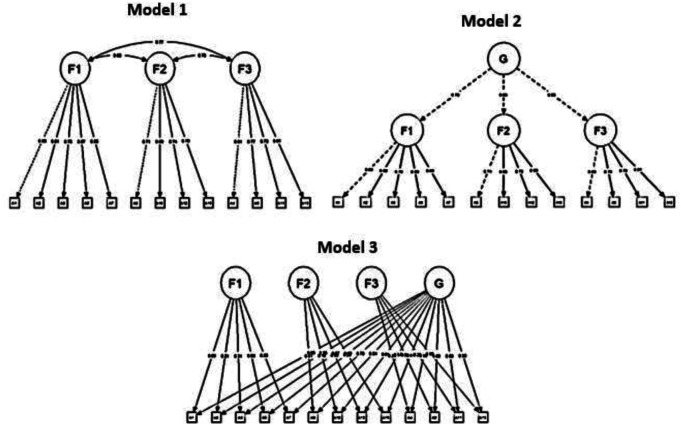
Factorial Models evaluated, three models were tested to determine the measurement model that best fit the data. Note: Model 1 = Correlated factors; Model 2 = Second order; Model 3 = Two factors; G = General Factor; F1 = Factor 1; F2 = Factor 2; F3 = Factor 3.

Figure 1 shows the three specified models that were tested to determine the best measurement model to fit the data: Model 1 = Correlated factors, Model 2 = Second order, Model 3 = Two-factor. However, the decision on the most plausible structure was made following not only statistical, but also theoretical, methodological, and ultimately practical criteria.

Table 3 shows that the three proposed models obtained adequate fit indexes: CFI ≥ .94, TLI ≥ .94, SRMR ≤ .08, RMSEA ≤ .07. Although they present differences, they are not relevant. However, in mathematical terms, it is clear that model 3 shows better relative fit indexes (χ ^2^/gl = 4.95 < 5). Therefore, the specific indexes for a bifactor model were further examined. The conjunction of the values was as follows: the hierarchical omega coefficient for the general factor (ωH > .80), the hierarchical omega coefficient for the specific factors (ωhs ≥ .30), the construct replicability coefficient (H > .90), the common variance explained (ECV > .60), and the percentage of uncontaminated correlations (PUC < .80) allow us to affirm the existence of a general factor that empirically justifies obtaining an overall score for the DAS Scale, with a proportion of explained variance of 69.56%. Finally, it is also noteworthy that the data obtained for the death anxiety variable, considering this bifactor model, is highly reliable for multidimensional scales (ω ≥ .80). Thus, the evidence indicates that the structure of three specific factors and one general factor is the most plausible. Moreover, it is consistent with the theory, is derived from the analysis methodology applied, and has practical utility, in that it would allow in the future to determine the greater weight of any of these three factors in the anxiety responses of the examinees.

**Table 3.** Confirmatory factor analysis of the DAS.

Models	χ ^2^	gl	χ ^2^/gl	CFI	TLI	RMSEA [IC 90%]	SRMR
Model 1: Correlated factors	473.553	62	7.638	.964	.955	.073 [.067;.079]	.048
Model 2: Second order	429.783	64	5.638	.968	.961	.068 [.062;.074]	.050
Model 3: Bifactorial	257.369	52	4.949	.982	.973	.056 [.050; .063]	.036

Ítems	Second-order model				Bifactor model			
	*λF*G	*λ*F1		*λF*G	*λ*F1		*λF*G	*λ*F1
A1	.629	.496	−	−	.632	.596	−	−
A2	.485	.382	−	−	.561	.209	−	−
A3	.599	.472	−	−	.667	.337	−	−
A5	.697	.549	−	−	.691	.58	−	−
A7	.676	.532	−	−	.789	.281	−	−
A8	.629	−	.387	−	.538	−	.610	−
A10	.577	−	.355	−	.609	−	.161	−
A12	.620	−	.381	−	.571	−	.531	−
A15	.591	−	.364	−	.608	−	.253	−
A4	.629	−	−	.202	.587	−	−	.238
A6	.727	−	−	.234	.692	−	−	.513
A11	.689	−	−	.221	.665	−	−	.252
A13	.577	−	−	.185	.58	−	−	.088
*ω*	.926	.892	.802	.784	.929	.893	.805	.790
*ω _H_*	.817	.342	.220	.073	.834	.236	.249	.124
*H*	.898	.617	.392	.158	.903	.568	.52	.330
*ECV*	.726	−	−	−	.717	−	−	−
*PUC*	.718	−	−	−	.718	−	−	−
% Explained variance	66.75	11.70	4.84	0.53	69.56	5.57	6.20	1.54

Note: F1 = Fear of death, F2 = Fear of life coming to an end, F3 = Fear of dying or illness, G = Death anxiety, λ= Standardized factor loadings; all associated p sig. chi-square and factor loadings were statistically significant at the .05 level.

### 3.3 Factorial invariance

As Table 1 shows, for the most part, the DAS items meet the parameters previously established to claim that the data matrix can be reduced to a smaller set of latent factors: magnitudes.

Finally, Table 4 presents the results of the factorial invariance analysis as a function of sex and age. Along these lines, the magnitudes of the variations in CFI (ΔCFI < .010), RMSEA (ΔRMSEA < .015), and SRMR (ΔSRMR ≤ .030) were minimal. Thus, the empirical evidence shows that the study participants interpret the meaning of the DAS items in a very similar way and that the scores obtained in this test are only attributable to the presence of the latent variable death anxiety, without depending on the characteristics of the examinees. Therefore, the new version of the DAS measures death anxiety fairly, without bias, in the Peruvian adult population, regardless of gender or age.

**Table 4.** Factorial invariance analysis as a function of sex and age group.

Sex (Men = 653, Women = 595)										
Levels	χ ^2^	Δχ ^2^	gl	Δgl	CFI	ΔCFI	RMSEA	ΔRMSEA	SRMR	ΔSRMR
Configural	349.744	−	104	−	.979	−	.062	−	.043	−
Metric	32.195	29.549	126	22	.984	.004	.050	.012	.048	.005
Strong	393.091	72.895	148	22	.979	.004	.052	.002	.046	.002
Strict	393.091	.000	148	0	.979	.000	.052	.000	.046	.000

Age group (Young = 863, Adults = 375)										
	χ ^2^	Δχ ^2^	gl	Δgl	CFI	ΔCFI	RMSEA	ΔRMSEA	SRMR	ΔSRMR
Configural	317.313	−	104	−	.983	−	.057	−	.040	−
Metric	39.877	73.564	126	22	.979	.004	.058	.001	.050	.010
Strong	395.699	4.822	148	22	.980	.001	.052	.006	.043	.007
Strict	395.699	.000	148	0	.980	.000	.052	.000	.043	.000

Note: Δχ ^2^ = changes in χ ^2^ test, Δgl = changes in degrees of freedom, ΔCFI = changes in CFI, ΔRMSEA = changes in RMSEA, ΔSRMR = changes in SRMR; * Probability value is statistically significant (p < .05).

## 4. Discussion

This study is the first to examine the psychometric properties of the Death Anxiety Scale and to provide evidence of its usefulness as a measure of this variable in the general non-clinical population of Peru (n=1248). Studies with a clinical sample were only conducted in Trujillo, ^17^ health personnel in Lima, ^18^ Trujillo, older adults ^19^ and older adults in Chimbote. ^20^ All of them were in zones and had samples of less than 600 subjects. It should be noted that the reliability for internal consistency in health personnel reached .95, followed by older adults in Trujillo ^19^ with .93. In contrast, in other countries such as Argentina ^16^ in 859 adult subjects, it only reached a reliability of .80, and in Spain ^15^ in a clinical sample, the value was .77.

To perform the CFA, three measurement models were tested to perform CFA, which is a good option when validating instruments with confirmatory factor analysis. ^54^ Initially, a model of three correlated factors was tested, as proposed by Rivera-Ledesma (2009) ^12^ in its Mexican version, as it is the version with the largest number of recent studies in Spanish. Subsequently, two hierarchical models were tested: a second-order formative model and another bifactor multidimensional model, in order to find empirical evidence in favor of the existence of a global score on the Death Anxiety Scale.

Consequently, our results support the three-dimensional structure of the DAS, which in turn allows a global score, as reported in previous national ^18^^–^^20^ and international studies, ^12^ in Spain, four factors were found in a clinical sample, ^15^ whereas in Argentina, in non-clinical adults, a better fit to a unifactorial structure was found. This indicates that the DAS, at least in the Peruvian context, can be used as a self-report instrument to measure death anxiety in the general nonclinical adult population.

### 4.1 Psychometric properties of the DAS

The construct validity of the DAS was tested and found to be satisfactory based on the CFA, and all model fit indices were acceptable. The AFC showed that the model that best fits our data was the three-factor oblique correlated model for all the three dimensions. The three-factor oblique model was better than the one- and two-factor models, indicating that death anxiety has a total score as a variable and, in turn, is composed of three dimensions.

We chose to eliminate two items with poor factor loadings (factor loadings < 0.4) and weaker values than other items in the study sample, such as item 9 (“I am afraid of having a painful death”) of the fear of dying dimension. Likewise, Item 14 (“I am horrified to see a corpse”) of the dimension of fear of life coming to an end. Thus, the adequacy of the model is improved.

The internal consistency reliability results of the DAS showed excellent values for all dimensions, with a Cronbach’s alpha and McDonald’s Omega ≥ .80. These results confirmed previous reports on the reliability of DAS in different studies and populations. ^12^^–^^20^

### 4.2 Measurement invariance

In the present study, the fairness of measurement between sexes and age was also examined using a factorial invariance analysis. It was found that the DAS exhibits complete configurational and metric invariance between sexes and ages. This indicates that DAS can be used to measure death anxiety with the same meaning for women and men or for Peruvian adult subjects of different non-clinical ages. That is, there were no differences in the structure of DAS between the sexes and age groups in the study sample. This result disagrees with the findings of Peñaloza et al., ^19^ who reported that scores differed between men and women.

### 4.3 Strengths and limitations

The main contribution of this study is that it is the first time that DAS validation has been performed in the general Peruvian population, covering most regions of Peru. However, this study had some limitations. First, a cross-sectional and not longitudinal design was used, in which the reliability analysis was based on internal consistency and not on the test-retest stability measure. Second, although death anxiety was universal, Non-probability sampling could pose a limitation in generalizing the sample results to the population. Third, the use of this type of convenience sampling could cause selection bias since only subjects with access to social networks responded to the online questionnaire. Finally, given that the general population was included, other limitations were that we did not analyze religious practice or the degree of religious involvement and background of chronic disease, which will be options for future studies.

## 5. Conclusions

In summary, our results show that the DAS has good psychometric properties for Peruvian adults. We found that DAS is valid and reliable, with the ability to clearly distinguish death anxiety from other factors. Our data suggest that this instrument is invariant between sexes and non-clinical age groups. Consequently, DAS can be used as a self-report instrument to detect death anxiety in the general adult Peruvian population. This will allow public policies, such as assistance programs, to identify subjects at higher risk and more vulnerable to developing mental health disorders related to quality of life that the state should guarantee.

This study is framed in the field of public and mental health, specifically constituting a methodological contribution that will overcome the lack of a useful, valid, and reliable instrument in the current Peruvian context, involving much more accurate and rigorous measurements of the variable in question, thus serving not only health professionals but also promoters and citizens themselves.

All of these factors contribute to the challenge of preventing and reducing the risk of mental diseases and achieving a biopsychosocial balance. This perspective is in line with the framework of the Global Goals of the 2030 Agenda for Sustainable Development, where Goal 3 aims to ensure healthy lives and promote well-being for all ages worldwide.

## Consent statement

The consent of the participants was given in writing before answering the online instruments.

## Data Availability

Zenodo: De La Cruz-Valdiviano CB: **Death anxiety scale (DAS) /database**. Zenodo; 2023. doi: https://doi.org/10.5281/zenodo.10689053. ^73^ This project contains the following underlying data: Database (Statistical results after the application of the Death anxiety scale-DAS).xlsx
•
https://doi.org/10.5281/zenodo.10689053
•
https://datos.ucv.edu.pe/handle/20.500.14413/3 Zenodo: Death anxiety scale (DAS): internal structure and factorial invariance in Peruvian adults. Zenodo; 2024. https://doi.org/10.5281/zenodo.12191803. ^74^ This project contains the following extended data:
• Final results (word).docx • Ethics Committee Approval.pdf (Etica UCV 70 De La Cruz Valdiviano) • Permission to use the Death Anxiety Scale_De la Cruz. • Informed consent • Death anxiety in Peruvian adults during the COVID-19 pandemic (Open Access). Data are available under the terms of the Creative Commons Attribution 4.0 International license (CC-BY 4.0).

## References

[1] Limonero JT Tomás-Sábado J Fernández-Castro J : Competencia personal percibida y ansiedad ante la muerte en estudiantes de enfermería [Perceived personal competence and death anxiety in nursing students]. *Ansiedad y estrés.* 2010;16(2):177–188. Reference Source

[2] Tomás-Sábado JT Gómez-Benito J : Variables relacionadas con la ansiedad ante la muerte [Variables related to death anxiety]. *Rev Psicol Gen Apl.* 2003;56(3):257–279. Reference Source

[3] Sevilla-Casado M Ferré-Grau C : Ansiedad ante la muerte en enfermeras de Atención Sociosanitaria: datos y significados [Death anxiety in health and social care nurses: data and meanings]. *Gerokomos.* 2013;24(3):109–114. 10.4321/s1134-928x2013000300003

[4] Organización Panamericana de la Salud-OPS/OMS: La OMS caracteriza a COVID-19 como una pandemia WHO [characterizes COVID-19 as a WHO pandemic].(accessed on 17 January 2023). Reference Source

[5] Statista: Coronavirus: número acumulado mundial de casos 2020- 2022 [Coronavirus: global cumulative number of cases 2020-2022].(accessed on 19 January 2023). Reference Source

[6] Organización Panamericana de la Salud-OPS/OMS: La pandemia de COVID-19 aumenta en un 25% la prevalencia de la ansiedad y la depresión en todo el mundo COVID-19 [pandemic increases worldwide prevalence of anxiety and depression by 25%].(accessed on 17 January 2023). Reference Source

[7] Organización de las Naciones Unidas-UNESCO: Nuevas publicaciones cubanas para enfrentar efectos de la COVID-19 sobre la Educación [New Cuban publications to address the effects of COVID-19 on Education].(accessed on 10 april 2023). Reference Source

[8] Loaiza MV : Los niveles de ansiedad de los jóvenes casi que se duplicaron durante el primer confinamiento por covid-19, según un estudio [Anxiety levels in young people nearly doubled during first covid-19 confinement, study finds].(accessed on 1 March 2023). Reference Source

[9] Tomás-Sábado J : Miedo y ansiedad ante la muerte en el contexto de la pandemia de la COVID-19 [Fear and anxiety about death in the context of the COVID-19 pandemic]. *Rev Enferm Salud Ment.* 2020;16:26–30. 10.5538/2385-703x.2020.16.26

[10] Grupo RPP : MINSA reportó 167 nuevos casos y 4 muertes por la COVID-19 en el último día [MINSA reported 167 new cases and 4 deaths from COVID-19 in the last day].(accessed on 1 march 2023). Reference Source

[11] Blanco TM : Ansiedad ante la muerte y factores de vulnerabilidad asociados en ofensores sexuales recluidos en el Centro de Atención Institucional Adulto Mayor [Death anxiety and associated vulnerability factors in sex offenders detained at the Centro de Atención Institucional Adulto Mayor]. Graduate Thesis in Psychology, Facultad de Ciencias Sociales, San José, Costa Rica, 11 January 2011.

[12] Rivera Ledesma A Montero López M : Propiedades Psicométricas de la Escala de Ansiedad ante la Muerte de Templer en sujetos mexicanos [Psychometric Properties of the Templer Death Anxiety Scale in Mexican Subjects]. *Diversitas.* 2010;6(1):135–140. (accessed on 1 Juanary 2023). 10.15332/s1794-9998.2010.0001.10 Reference Source

[13] Sharif Nia H Lehto RH Pahlevan Sharif S : A cross-cultural evaluation of the construct validity of Templer’s Death Anxiety Scale: A systematic review. *OMEGA- J Death Dying.* 2021;83(4):760–776. 10.1177/0030222819865407 31366310

[14] Instituto Nacional de Estadística e Informática – INEI: Censos Nacionales 2017: XII de Población, VII de Vivienda y III de Comunidades Indígenas [National Censuses 2017: XII Population Census, VII Housing Census and III Indigenous Communities Census]. Sistema de Consulta de Base de Datos.(accessed on 11 March 2023). Reference Source

[15] López-Castedo A González-Rodríguez R Vázquez Pérez R : Propiedades psicométricas del Death Anxiety Stait en pacientes con cardiopatía isquémica [Psychometric properties of the Death Anxiety Stait in patients with ischemic heart disease]. *Rev Esp Salud Pública.* 2020;93:1–9. Reference Source PMC11583031 31576814

[16] Resett S Kenserbaum M González Caino P : Validación Preliminar de la Escala de Ansiedad ante la Muerte de Templer en una Muestra Argentina [Preliminary Validation of the Templer Death Anxiety Scale in an Argentinean Sample]. *Psykhe (Santiago).* 2021;30(1). 10.7764/psykhe.2018.21923

[17] Requejo Chapilliquén M Sánchez Carbajal D : Ajuste mental y ansiedad ante la muerte en pacientes con cáncer terminal [neuroscience Mental Adjustment and Death Anxiety in Terminal Cancer Patients. neuroscience]. *J Neuro Pub Health.* 2021;1(2):53–61. 10.46363/jnph.v1i2.3 Reference Source

[18] Peñaloza S : Propiedades psicométricas de la Escala de Ansiedad ante la Muerte (DAS) de Templer, en personal de salud, Lima Metropolitana- 2021 [ Psychometric Properties of Templer’s Death Anxiety Scale (DAS) in health care personnel, Lima Metropolitana- 2021]. Licentiate thesis in Psychology, Universidad César Vallejo; Lima-Norte, Peru, February 2022. Reference Source

[19] Grauss MK : Propiedades Psicométricas de la Escala de Ansiedad ante la Muerte en pacientes adulto mayor de Instituciones de la Ciudad de Trujillo [Psychometric properties of the Death Anxiety Scale in elderly patients from institutions in the city of Trujillo]. Licentiate thesis in Psychology, Universidad César Vallejo; Trujillo, Peru, September 2016. Reference Source

[20] Rodriguez JE : Propiedades Psicométricas de la Escala de Ansiedad ante la Muerte en adultos mayores de Chimbote [Psychometric Properties of the Death Anxiety Scale in Chimbote’s older adults]. Licentiate thesis in Psychology, Universidad César Vallejo; Chimbote, Peru, February 2020. Reference Source

[21] Gürbüz A Yorulmaz O : Death anxiety in psychopathology: A systematic review. *Psikiyatr. Guncel Yaklasimlar.* 2024;16(1):159–174. 10.18863/pgy.1267748

[22] Abdel-Khalek AM Neimeyer RA : Death Anxiety Scale.In: Zeigler-Hill V Shackelford TK , editors. *Encyclopedia of Personality and Individual Differences.* Springer International Publishing;2017. 10.1007/978-3-319-28099-8_21-1

[23] RAE Diccionario de la Real Academia Española: Ansiedad [Anxiety].(accessed on 1 Juanary 2023). Reference Source

[24] Limonero J : Evaluación de aspectos perceptivos y emocionales en la proximidad de la muerte [Assessment of perceptual and emotional aspects in the proximity of death]. Doctoral thesis, Universitat Autònoma de Barcelona; España. 1994. Reference Source

[25] Hamilton MC : Diagnosis and rating of anxiety. *Br J Psychiatry.* 1969;3:76–79.

[26] Eysenck HJ : A theory of the incubation of anxiety/fear responses. *Behav. Res. Ther.* 1968;6(3):309–321. 10.1016/0005-7967(68)90064-8 Reference Source 5734549

[27] Spearman C : Correlations of sums and differences. *Br J Psychol.* 1913;5:417–426.

[28] Tamayo M : *Diccionario de la investigación científica [Dictionary of scientific research].* 2da. Edic. LIMUSA;2014;242.

[29] Arias FG : *El proyecto de investigación [The research project].* 6ta. Edic. Caracas: Episteme;2012;143.

[30] Hernández-Sampieri R Mendoza C : *Metodología de la investigación. Las rutas cuantitativa, cualitativa y mixta [Research methodology. Quantitative, qualitative and mixed routes].* 1ra. edic. DF: McGraw Hill, México;2018;714. Reference Source

[31] Comrey AL Lee HB : *A first course in factor analysis.* 2nd ed. Editorial Lawrence Erlbaum Associates, Inc;1992;442.

[32] Alarco JJ Álvarez EV : Google Docs: una alternativa de encuestas online [Google Docs: an online survey alternative]. *Educ Médica.* 2012;15(1):9–10. 10.4321/S1575-18132012000100004

[33] Miaja M : Propiedades psicométricas de la escala de ansiedad ante la muerte en personas con VIH y población general [Psychometric properties of the death anxiety scale in people with HIV and the general population]. *Rev Psicopatol y psicol Clín.* 2012;17(2):107–122. 10.5944/rppc.vol.17.num.2.2012.11208 Reference Source

[34] Zuccala M Menzies RE Hunt CJ : A systematic review of the psychometric properties of death anxiety self-report measures. *Death Stud.* 2022;46(2):257–279. 10.1080/07481187.2019.1699203 31809665

[35] World Medical Association (WMA): Declaration of Helsinki – ethical principles for medical research involving human subjects. (s/f). Recuperado el 25 de abril de 2024. Reference Source Reference Source

[36] Colegio de Psicólogos del Perú: Código de ética [Code of ethics].(accessed on 10 March 2023). Reference Source

[37] Revelle W : *Psych: Procedures for Psychological, Psychometric, and Personality Research.* Evanston, Illinois: Northwestern University; R package version 2.3.3, 2023. Reference Source

[38] Bandalos DL Finney SJ : Factor analysis: Exploratory and confirmatory. *The Reviewer’s Guide to Quantitative Methods in the Social Sciences.* 2nd ed. New York: Routledge;2018; pp.98–122.

[39] Muthén B Kaplan D : A comparison of some methodologies for the factor analysis of non-normal Likert variables. *Br J Math Stat Psychol.* 1985;38(2):171–189. 10.1111/j.2044-8317.1985.tb00832.x

[40] Kline PA : *Handbook of test construction: Introduction to psychometric design.* 1a ed. London, England: Routledge;2016.

[41] Williams B Onsman A Brown T : Exploratory factor analysis: A five-step guide for novices. *Australas J Paramed.* 2010;8(3):1–13. 10.33151/ajp.8.3.93 Reference Source

[42] Ferrando PJ Anguiano-Carrasco C : El análisis factorial como técnica de investigación en psicología [Factor analysis as a research technique in psychology]. *Pap Psicól.* 2010;31(1):18–33.

[43] Tabachnick BG Fidell LS : *Using multivariate statistics.* 7a ed. Upper Saddle River, NJ: Pearson;2018.

[44] Lüdecke D Ben-Shachar M Patil I : Extracting, computing and exploring the parameters of statistical models using R. *J Open Source Softw.* 2020;5(53):24–45. 10.21105/joss.02445

[45] López-Aguado M Gutiérrez-Provecho L : Cómo realizar e interpretar un análisis factorial exploratorio utilizando SPSS [How to perform and interpret an exploratory factor analysis using SPSS]. *REIRE Rev Innov Recer Educ.* 2019;12 (2)(2):1–14. 10.1344/reire2019.12.227057

[46] Lloret-Segura S Ferreres-Traver A Hernández-Baeza A : El análisis factorial exploratorio de los ítems: una guía práctica, revisada y actualizada. [Exploratory factor analysis of items: a practical guide, revised and updated]. *An Psicól.* 2014;30(3):51–69. 10.6018/analesps.30.3.199361 Reference Source

[47] Dominguez-Lara S Rodriguez A : Índices estadísticos de modelos bifactor [Statistical indices of bifactor models]. *Interacciones: Rev Av Psicól.* 2017;3(2):59–65. 10.24016/2017.v3n2.51

[48] Ferrando PJ Anguiano-Carrasco C : El análisis factorial como técnica de investigación en psicología [Factor analysis as a research technique in psychology]. *Pap Psicól.* 2010;31(1):18–33. Reference Source

[49] Méndez Martínez C Rondón Sepúlveda MA : Introducción al análisis factorial exploratorio [Introduction to exploratory factor analysis]. *Rev Colomb Psiquiatr.* 2012;41(1):197–207. 10.1016/S0034-7450(14)60077-9 Reference Source 26573478

[50] Rosseel Y : Iavaan: an R package for structural equation modeling. *J Stat Softw.* 2012;48(2):1–36. 10.18637/jss.v048.i02

[51] Dominguez Lara SA : Análisis psicométrico de la escala de bienestar psicológico para adultos en estudiantes universitarios de Lima: un enfoque de ecuaciones estructurales [Psychometric analysis of the psychological well-being scale for adults in university students in Lima: a structural equation approach]. *Psychol.* 2014;8(1):23–31. 10.21500/19002386.1211 Reference Source

[52] Brown TA : *Confirmatory factor analysis for applied research.* second edition. 2nd. edic. New York, NY: Guilford Publications;2014. Reference Source

[53] DiStefano C Morgan GB : A comparison of diagonal weighted least squares robust estimation techniques for ordinal data. *Struct Equ Modeling.* 2014;21(3):425–438. 10.1080/10705511.2014.915373

[54] Herrero J : El Análisis Factorial Confirmatorio en el estudio de la Estructura y Estabilidad de los Instrumentos de Evaluación: Un ejemplo con el Cuestionario de Autoestima CA-14 [Confirmatory Factor Analysis in the Study of the Structure and Stability of Assessment Instruments: An Example with the Self-Esteem Questionnaire CA-14]. *Interv Psicosoc.* 2010;19(3):289–300. 10.5093/in2010v19n3a9 Reference Source

[55] Hair JF Black WC Babin BJ : *Multivariate data analysis.* 8th edic. Boston: Cengage;2019.

[56] Marôco J : *Análise de Equações Estruturais: fundamentos teóricos, software & aplicações [Structural Equation Analysis: theoretical foundations, software & applications].* 2nd. edic. ReportNumber;2014.

[57] Schmid J Leiman JM : The development of hierarchical factor solutions. *Psychometrika.* 1957;22(1):53–61. 10.1007/bf02289209

[58] Wolff HG Preising K : Exploring item and higher order factor structure with the Schmid-Leiman solution: syntax codes for SPSS and SAS. *Behav Res Methods.* 2005;37(1):48–58. 10.3758/bf03206397 16097343

[59] Steiner M Grieder S : EFAtools: An R package with fast and flexible implementations of exploratory factor analysis tools. *J Open Source Softw.* 2020;5(53):2521. 10.21105/joss.02521

[60] Reise SP Scheines R Widaman KF : Multidimensionality and structural coefficient bias in structural equation modeling: A bifactor perspective. *Educ Psychol Meas.* 2013;73(1):5–26. 10.1177/0013164412449831

[61] Smits IA Timmerman ME Barelds DP : The Dutch symptom checklist-90-revised. *Eur J Psychol Asess Assessment.* 2014;31(4):263–271. 10.1027/1015-5759/a000233

[62] Hancock GR Mueller RO : Rethinking construct reliability within latent variable systems. Cudeck R Toit S du Sörbom D , editors. *Structural equation modeling: Present and future—A Festschrift in honor of Karl Jöreskog.* Scirp.org;2001; pp.195–216. Reference Source

[63] McDonald RP : *Test theory: A unified treatment.* 1st ed. London, England: Psychology Press;2013. 10.4324/9781410601087

[64] Dunn TJ Baguley T Brunsden V : From alph t omega: A practical solution to the perva-sive problem of internal consistency estimation. *Br J Psychol.* 2013;105(3):399–412. 10.1111/bjop.12046 24844115

[65] Rodriguez A Reise SP : Haviland MG Applying bifactor statistical indices in the evaluation of psychological measures. *J Pers Assess.* 2015;98(3):223–237. 10.1080/00223891.2015.1089249 26514921

[66] Nunnally JC : An overview of psychological measurement. Clinical diagnosis of mental disorders: A handbook. 1978;97–146.

[67] Byrne BM : Testing for multigroup equivalence of a measuring instrument: a walk through the process. *Psicothema.* 2008;20(4):872–882. Reference Source 18940097

[68] Jorgensen TD : How to estimate absolute-error components in structural equation models of generalizability theory. *Psych.* 2021;3(2):113–133. 10.3390/psych3020011 Reference Source

[69] Chen FF : Sensitivity of goodness of fit indexes to lack of measurement invariance. *Struct Equ Modeling.* 2007;14(3):464–504. 10.1080/10705510701301834 Reference Source

[70] Cheung GW Rensvold RB : Evaluating goodness-of-fit indexes for testing measurement invariance. *Struct Equ Modeling.* 2002;9(2):233–255. 10.1207/s15328007sem0902_5

[71] Hirschfeld G Von Brachel R : Improving Multiple-Group confirmatory factor analysis in R–A tutorial in measurement invariance with continuous and ordinal indicators. *Pract Assess Res Eval.* 2014;19(1):7. Reference Source

[72] Dimitrov DM : Testing for factorial invariance in the context of construct validation. *Meas Eval Couns Dev.* 2010;43(2):121–149. 10.1177/0748175610373459

[73] De La Cruz-Valdiviano CB : Death anxiety scale (DAS) /database. *Zenodo.* 2023. 10.5281/zenodo.10689053 PMC11621609 39649839

[74] De La Cruz-Valdiviano CB : Death anxiety scale (DAS): internal structure and factorial invariance in Peruvian adults.[Data set]. 10.5281/zenodo.12191803 PMC11621609 39649839

