# Supplementary material for: Death anxiety scale (DAS): internal structure and factorial invariance in Peruvian adults
Source: F1000Res. 2024 Nov 25;13:801. Originally published 2024 Jul 16. [Version 2] doi: 10.12688/f1000research.143167.2 (PMC11621609; doi:10.12688/f1000research.143167.2)
Supplement: Peer review history

# Reviewer report: Reviewer response for version 2

**DOI:** 10.5256/f1000research.174784.r343216

**Published:** 2024-12-05

**Authors:** Afreen Faiza

**Affiliations:** 1 Department of Psychology, Fatima College of Health Science, Abu Dhabi, United Arab Emirates

Article addressed all suggestions.

Is the work clearly and accurately presented and does it cite the current literature?

Yes

If applicable, is the statistical analysis and its interpretation appropriate?

Yes

Are all the source data underlying the results available to ensure full reproducibility?

Yes

Is the study design appropriate and is the work technically sound?

Partly

Are the conclusions drawn adequately supported by the results?

Yes

Are sufficient details of methods and analysis provided to allow replication by others?

Partly

Reviewer Expertise:

Psychometrics, thanatology, theology

I confirm that I have read this submission and believe that I have an appropriate level of expertise to confirm that it is of an acceptable scientific standard.

# Reviewer report: Reviewer response for version 1

**DOI:** 10.5256/f1000research.156799.r328813

**Published:** 2024-10-22

**Authors:** Hanaa Shuwiekh

**Affiliations:** 1 Fayoum University, Faiyum, Faiyum Governorate, Egypt

**Good research in its methodological treatment, although the concept, which is death anxiety, has been studied a lot. Good luck.**

Is the work clearly and accurately presented and does it cite the current literature?

Yes

If applicable, is the statistical analysis and its interpretation appropriate?

Yes

Are all the source data underlying the results available to ensure full reproducibility?

Yes

Is the study design appropriate and is the work technically sound?

Yes

Are the conclusions drawn adequately supported by the results?

Yes

Are sufficient details of methods and analysis provided to allow replication by others?

Yes

Reviewer Expertise:

NA

I confirm that I have read this submission and believe that I have an appropriate level of expertise to confirm that it is of an acceptable scientific standard.

# Reviewer report: Reviewer response for version 1

**DOI:** 10.5256/f1000research.156799.r304672

**Published:** 2024-08-12

**Authors:** Afreen Faiza

**Affiliations:** 1 Department of Psychology, Fatima College of Health Science, Abu Dhabi, United Arab Emirates

1. Literature Review Section could include recent findings related to death anxiety and its components focused on death anxiety in context of general population as the sample target for scale validation seems general population rather than terminal or physically ill or preoccupation about death. 

 2. Do  Studies cited in : Section 1.1

 Rivera and Montero ^12^ Peñaloza ^18 ^used Templar DAs?? if yes please state. 

 3. do we really need to write separately about anxiety and make another section 1.2 ?  better link it with death anxiety or write description about how fear is different from anxiety. 

 4.  what was religious backgrounds/ health status  of participants,  

 5. major concern is that how templer DAS divided into 3 factors ?? what was your rationale to do so as it  has one pf major drawback was that TDAs is uni dimensional scale. 

 how we come up with different names of sub scales : **Fear of agony or pathology, Fear of life coming to an end, Fear of death,**

 ( what subscales measures, and how it comply with theory of Templer scale )

Is the work clearly and accurately presented and does it cite the current literature?

Yes

If applicable, is the statistical analysis and its interpretation appropriate?

Yes

Are all the source data underlying the results available to ensure full reproducibility?

Yes

Is the study design appropriate and is the work technically sound?

Partly

Are the conclusions drawn adequately supported by the results?

Yes

Are sufficient details of methods and analysis provided to allow replication by others?

Partly

Reviewer Expertise:

Psychometrics, thanatology, theology

I confirm that I have read this submission and believe that I have an appropriate level of expertise to confirm that it is of an acceptable scientific standard, however I have significant reservations, as outlined above.

# Response

**Published:** 2024-11-19

**Authors:** Carlos De La Cruz-Valdiviano

**Affiliations:** Lima, Cesar Vallejo University, Lima, Lima, Peru

1. Literature Review Section could include recent findings related to death anxiety and its components focused on death anxiety in context of general population as the sample target for scale validation seems general population rather than terminal or physically ill or preoccupation about death. 

**Response:**

 In the literature review, the initial focus was on research concerning death anxiety in clinical populations due to the influence these studies have had on the validation of the Death Anxiety Scale (DAS). However, we understand the importance of providing a more updated and relevant framework for the general population, as the sample in this study consists of Peruvian adults without severe medical conditions. To strengthen the literature review section, we have consulted recent studies addressing death anxiety in the general population, and I include below some relevant findings that will be integrated into the manuscript:

**Influence of Sociocultural Factors:** Recent research has indicated that death anxiety is strongly influenced by sociocultural and contextual factors ^72^ . These studies emphasize that death anxiety can vary significantly between countries and cultures, depending on elements such as religious beliefs, social norms, and exposure to conflict contexts. This aspect is particularly relevant for the Peruvian population, given the cultural diversity and sociocultural values that could influence levels of death anxiety.

**Death Anxiety in Young and Middle-Aged Adults:** Studies such as those by Abdel-Khalek and Neimeyer ^73^ have demonstrated that death anxiety is not exclusive to clinical populations or older adults. High levels of death anxiety have been observed in young and middle-aged adults, who face uncertainties common to this stage of life, such as changes in family and professional roles. These findings are crucial for the context of our Peruvian sample, as most participants fall within a similar age range.

**Gender Differences:** Some recent studies have suggested that there are significant gender differences in the manifestation of death anxiety in the general population ^73^ . Women tend to report higher levels of death anxiety than men, which could be attributed to a greater predisposition to experiencing emotional concerns. This aspect will be included in the review to provide better contextualization of the study's target population.

 Relationship between Death Anxiety and Psychological Well-Being: Lastly, recent studies have explored the relationship between death anxiety and psychological well-being in the general population. According to Abdel-Khalek and Neimeyer ^73^ , death anxiety is inversely related to subjective well-being, indicating that elevated levels of death anxiety are associated with a decrease in quality of life. This finding is particularly relevant to emphasize the importance of adequately measuring death anxiety in the general population and considering its implications for mental well-being.

 We once again appreciate your exhaustive review and your suggestions, which will undoubtedly enrich the scope and depth of the analysis of this study. However, we believe that framing the research in the context of the pandemic justifies studying death anxiety in the general population.

 2. Do  Studies cited in : Section 1.1 Rivera and Montero ^12^ Peñaloza ^18^ used Templar DAs?? if yes please state

 Response: Yes, both cited studies used Templer's Death Anxiety Scale (DAS) as an instrument to assess death anxiety in their respective samples. Specifically, the study by Rivera and Montero evaluated the psychometric properties of the DAS in a sample of Mexican subjects, while Peñaloza's study also used Templer's DAS to examine its psychometric properties in a sample of healthcare personnel from Lima Metropolitan.

 3. Do we really need to write separately about anxiety and make another section 1.2 ?  better link it with death anxiety or write description about how fear is different from anxiety. 

**Response:**

 We understand the reviewer's concern regarding the separation of the sections related to anxiety and death anxiety. However, we believe that the way these sections were organized has solid theoretical and methodological support, which also reflects a logical and appropriate structure for the reader's understanding.

 First, in the section on anxiety (1.2), the intention was to provide a conceptual framework necessary to understand anxiety from a general perspective. Anxiety is a central component in analyzing death anxiety, but each has specific characteristics that needed to be explained independently to avoid confusing the reader. Anxiety is a state of general arousal that can manifest in different contexts and be influenced by various factors, such as existential fear and the perception of the threat of death ^74^ . Separating these concepts allowed us to clarify the distinction between anxiety as a broad phenomenon and death-related anxiety, thereby adhering to an academic rigor criterion that facilitates a comprehensive understanding of the construct of death anxiety.

 Second, the inclusion of a section discussing general anxiety is also justified from the perspective of Terror Management Theory (TMT), as presented by Zuccala et al. ^75^ . This theory proposes that death anxiety is a specific form of anxiety underlying various mental disorders, and its correct understanding begins by defining anxiety as a general response to the perception of existential threats. This approach allowed us to develop a solid conceptualization of how fear of death is linked to other fears and anxieties, and laid the foundation for the clear differentiation between fear and anxiety described later.

 Third, the structure chosen for the manuscript also responds to methodological coherence, as the factorial solutions found in the exploratory and confirmatory analysis revealed three distinct dimensions in Templer's Death Anxiety Scale (DAS). Each of these dimensions is linked to specific components of anxiety that, according to studies such as those by Sharif Nia et al. ^74^ , differ significantly in different cultural and sociodemographic contexts. The presence of multiple factors suggests that death anxiety is not merely a unidimensional fear but can be expressed in different forms, which justifies the proposed structure.

 Finally, we consider that the preprint publication of the manuscript already reflects a consensus on the organization of the sections and the methodological decisions made, which have already been reviewed and accepted (100%) by one of the peers. The need to discuss anxiety in general terms before addressing death anxiety is also a response to previous studies that emphasize the importance of contextualizing and clearly defining these concepts to ensure a precise understanding of the construct ^75^ .

 For these reasons, we believe that it is not necessary to make modifications to the manuscript's structure. The current organization allows for a better understanding of the relationship between anxiety and death anxiety, meeting criteria for clarity and methodological rigor that are essential in this type of study.

 4.  what was religious backgrounds/ health status  of participants?

**Response:**

 Regarding the religious backgrounds of the participants, we acknowledge that this information represents one of the limitations of the present study. Detailed data on religious affiliation or the degree of religious involvement of the participants were not collected. It is important to mention that this aspect may have influenced the interpretation and response of participants to the Death Anxiety Scale (DAS), as religion can act as a modulatory mechanism of the fear of death. Consequently, this limitation is highlighted in the discussion of the study, and we suggest that future research include a thorough analysis of the impact of religious backgrounds to gain a deeper understanding of the phenomenon of death anxiety.

 Concerning health status, the study focused on the history of COVID-19, given the relevance of the pandemic to the experience of death anxiety in the general population. Specific data were collected on whether participants had contracted the disease, and the results showed that 45% of respondents reported having had COVID-19. This variable was considered due to the known impact of the pandemic on perceptions of vulnerability and the increase in existential anxiety in many people. However, other aspects of general health, such as the presence of chronic or terminal illnesses, were not addressed, which also constitutes a limitation of this study. This information will be included more clearly in the discussion to indicate how these limitations may have influenced the results obtained and how they should be addressed in future research.

 I once again appreciate your careful review and suggestions, which undoubtedly enrich the scope and depth of this study's analysis. Nevertheless, I believe that framing the research within the context of the pandemic justifies studying death anxiety in the general population.

 5. Major concern is that how templer DAS divided into 3 factors ?? what was your rationale to do so as it  has one pf major drawback was that TDAs is uni dimensional scale.  how we come up with different names of sub scales :  Fear of agony or pathology, Fear of life coming to an end, Fear of death, ( what subscales measures, and how it comply with theory of Templer scale?)

**Response:**

**Justification of the Factor Structure**

 In our study, the Death Anxiety Scale (DAS) was subjected to exploratory factor analysis (EFA) and confirmatory factor analysis (CFA), revealing a structure of three interrelated factors, which contradicts the traditionally unidimensional nature attributed to the scale. These three emerging factors were named: Fear of agony or pathology, Fear of life ending, and Fear of death. The results indicated an accumulated variance of 53.3%, which supports the existence of additional components beyond the general factor of death anxiety.

**Naming the Subscales**

 Fear of agony or pathology: This factor includes items that reflect concern about physical suffering or pain associated with death. The naming is derived from the qualitative analysis of the content of the items that make up this factor, which focus on aspects of distress over a painful death or prolonged illness. These findings are consistent with previous findings by Rivera and Montero, as well as Peñaloza, who also found that fear of the process of agony is a crucial component in the structure of death anxiety.

 Fear of life ending: This factor represents existential fear related to the perception of the end of life as an inevitable process. The items associated with this component suggest a concern for the finite nature of life and the uncertainty that comes with it, clearly distinguishing it from physical fear or the consequences of death. This type of fear has also been documented in cross-cultural studies where differences are observed in how the end of life is perceived and feared depending on the cultural context and sample evaluated ^74^ .

 Fear of death: This factor groups items that address the fear of the idea of death itself, including the fear of the unknown that death represents. This component relates to abstract or philosophical anxiety about the act of dying, which is consistent with studies that have evidenced a distinct psychological component in death anxiety ^75^ . In the cross-cultural analysis by Sharif Nia et al. ^74^ , it was observed that the factor structure of the DAS varied significantly across different cultural contexts, highlighting the need to adapt the scale according to the specific characteristics of each population.

**Coherence with Templer's Theory**

 Although Templer's Scale was initially conceived as unidimensional, recent studies have suggested the existence of multiple dimensions depending on the cultural and population context in which it is applied. For instance, the systematic review conducted by Sharif Nia et al. ^74^ found factor structures ranging from two to five factors, depending on the cultural context and evaluated population. This evidence supports the need to reconsider the unidimensionality of the scale in specific contexts like the Peruvian one, in which we identified three factors that seem to capture particular nuances of death anxiety. Zuccala et al. ^75^ also point out that variability in the DAS factor structure can be attributed to cultural differences and the translation of the instrument's content. Indeed, death anxiety tends to manifest differently according to the cultural context, directly affecting the scale's validity and reliability. Therefore, it has been recommended to conduct continuous evaluations and specific cultural adaptations before applying it in clinical and multicultural settings.

**Empirical Evidence**

 In the confirmatory factor analysis (CFA), the bifactor model best fit the data, with adequate fit indices (CFI ≥ .94, TLI ≥ .94, SRMR ≤ .08, RMSEA ≤ .07) and a proportion of explained variance of 69.56%. This suggests that while a general factor measuring death anxiety exists, the three specific dimensions have sufficient independence to be considered validated subscales, providing greater precision in the evaluation of death anxiety. This is consistent with the results of the exploratory factor analysis previously mentioned.

**Conclusion**

 The division of the Death Anxiety Scale into three factors is based on rigorous empirical analysis that reflects the underlying structure of the data and is in line with previous cross-cultural studies that have identified multidimensional structures. This conceptualization allows for a more comprehensive and nuanced assessment of death anxiety in the Peruvian population, capturing specific aspects that may not be evident in a unidimensional structure. The results of Sharif Nia et al. ^74^ and Zuccala et al. ^75^ support the need to adapt the DAS scale according to the cultural and sociodemographic particularities of each sample, which motivated us to validate a three-dimensional structure in the Peruvian context to provide a more sensitive and accurate assessment tool for our target population.
